# A Plea for Accuracy of Thought in Medicine

**Published:** 1907-08-03

**Authors:** 


### A Plea for Accuracy of Thought in Medicine.

We will venture to congratulate Dr. Hale White on the fortunate and appropriate inspiration which prompted him to present as his official address to the British Medical Association "A Plea for Accuracy of Thought in Medicine." The occasion is one which has claims that are not readily satisfied. In the present instance it will be readily recognised that the speaker has fully appreciated his responsibilities, and has affirmed and defended propositions that need careful consideration in the interests both of the profession and of the public. That he has departed from the easy habit of prophesying smooth things may be accepted not without gratitude, and if some of us feel our ears tingling from his words, our true interests may yet recognise themselves as substantially in his debt. In any event, he has spoken manfully and with good intent, and he deserves the recognition due to those who discharge duty with a calm and considered courage.

The plea for accuracy in thought and speech which the address advances is a plea which no one will resist. In theory, at all events, it commands universal assent. This, of course, is as true of the general affairs of life as it is of the work of the practitioner of medicine. And it is from this universally recognised position that Dr. Hale White starts to examine how far practice in this respect consorts with theory, and to show what disastrous results accumulate when the two are separated. His conclusion quite frankly expressed is that the medical profession over large areas of its activity is content with a loose and inaccurate method of thought and speech, and that only too often it is satisfied with words and phrases which cannot be said to possess a corresponding basis of fact. To this main indictment we fear a verdict of "Guilty" must be entered. It is without question a common custom to pronounce that a bronchitis or a sciatica or an irregular action of the heart is "gouty," when, in the individual case concerned, there is no scrap of evidence to justify the conclusion so expressed. Similarly the liver is repeatedly made to carry a diagnostic burden altogether disproportionate to the justification provided by precise pathological knowledge. And what applies to these particular instances may be extended widely over the entire field of medical practice. Names and phrases which carry a confident sound are freely employed, though a critical examination would prove them either to be empty of meaning or to represent mere vague and unwarranted conjectures. It is well to have these things stated plainly and by those who occupy high places. We agree with Dr. Hale White that these loose habits of thought and speech are not merely bad in themselves, but that they constitute a very real and substantial barrier to the progress of medical knowledge. Words without knowledge, we are told, darken counsel; and, still worse, they lead to a cheap and self-satisfied content, which is the enemy of all effective progress.

The habit of precision of thought and statement, it may be urged, is at least as frequent in medical practice as it is in social life. In the ordinary affairs of the world custom appears to sanction the continual use of terms which have little justification other than that they save "talkative people the trouble of thinking." Yet it must be remembered that medicine claims a scientific basis, and the standard of scientific accuracy is fortunately somewhat higher than that of the drawing-room and the debating hall. Clear thinking, and a resolute determination to abstain from the profession of opinions without the warrant of facts, are commonplace canons of scientific discussion, and they are needed in medicine not less than in other departments of science. Again, it may be contended that the public expects the medical man to come to a prompt and certain conclusion even though the evidence is incomplete, and that a confession of ignorance is fatal to the retention of the confidence of patients. But, as Dr. Hale White well points out, the profession has in this respect too often been the mentor of the public; and mere verbal diagnoses and opinions content the laity, because, in the first place, they have been used to content ourselves. But it is well that we should be exhorted to reduce inaccuracy to a minimum, and that our own minds should be kept free from the delusion that to talk about a subject is to explain it. It was a great physician who said "Of all the gifts granted to man accuracy is the rarest." But here, as elsewhere, "who aimeth at the sky shoots higher far than he that means a tree."

